# Editorial: Acute and hereditary coagulation disorders

**DOI:** 10.3389/fmed.2024.1507527

**Published:** 2024-10-23

**Authors:** Herbert Schöchl, Felix Schmitt, Wolfgang Miesbach

**Affiliations:** ^1^Ludwig Boltzmann Institute for Traumatology, Salzburg, Austria; ^2^Heidelberg University, Heidelberg, Baden-Württemberg, Germany; ^3^University Hospital Frankfurt, Frankfurt, Germany

**Keywords:** editorial, advantages, coagulation, disorders, recent

We are pleased to present this edition on Research Topics about acute and hereditary coagulation disorders in “*Frontiers in Medicine*”.

Coagulation disorders remain a critical area of medical research. The field of coagulation disorders continues to evolve rapidly, with new insights emerging across various conditions. In this edition, the authors of several groundbreaking studies have advanced our understanding of coagulation disorders, from rare genetic conditions to common complications of pregnancy and critical illness. Their work spans a wide range of topics, including novel diagnostic techniques, treatment strategies, and prognostic markers.

Gruneberg et al. investigated the role of fibrinolytic potential in postpartum hemorrhage (PPH) in their study “*Fibrinolytic potential as a risk factor for postpartum hemorrhage*”. They found that women experiencing severe PPH showed increased fibrinolytic potential as early as hospital admission. This suggests that elevated fibrinolytic potential could be a risk factor for PPH, although additional factors are likely required to trigger the condition.

Alesci, Hecking et al. explored the utility of ACMG classification in factor VII deficiency in their study “*Utility of ACMG classification to support interpretation of molecular genetic test results in patients with factor VII deficiency*”. Their findings suggest that specific combinations of ACMG-classified variants correlate with high-risk bleeding phenotypes, potentially paving the way for genotype-phenotype prediction models in the future.

Navaei et al. addressed the question “*Is it time to switch to bivalirudin for ECMO anticoagulation?*”. While some studies showed the advantages of bivalirudin over heparin in preventing major bleeding and thrombosis, the majority found no significant difference. Further prospective studies are needed to reach a definitive conclusion.

Toenges et al. investigated acquired dysfibrinogenemia in septic patients in their study “*Investigation of acquired dysfibrinogenemia in adult patients with sepsis using fibrinogen function vs. concentration ratios: a cross-sectional study*”. Their results suggest the presence of acquired dysfibrinogenemia in some adult septic patients, potentially contributing to a specific laboratory signature of sepsis-associated coagulation phenotype.

Huang et al. examined the “*Current status of treatment and disease burden of a cohort of hemophilia B in China*”. They found that the use of prophylaxis was low, and patients faced significant financial burdens. The study highlights the need for improved access to safer and more effective drugs and efforts to reduce the financial burden on patients.

Quintana-Diaz et al. developed a mnemonic device “*COAGULATION*” for treating coagulation disorders following traumatic brain injury. This simple tool could aid clinicians in managing patients with moderate or severe traumatic brain injury on a daily basis.

Henze et al. conducted a prevalence study on “*Abdominal venous thromboses: detection of the JAK2 p.V617F mutation by next-generation ultradeep sequencing*”. They found the JAK2 p.V617F mutation in 19% of patients with abdominal venous thromboses, highlighting the importance of genetic analysis in understanding the etiology of these rare thrombotic events.

Alesci, Goldmann et al. surveyed patients with mild hemophilia in Germany to gain insights into their treatment reality and quality of life. Their findings suggest that this population may underestimate bleeding complications, emphasizing the need for improved awareness and treatment strategies.

Goodarzi et al. compared two fibrinogen concentrates in their case report “*Are all fibrinogen concentrates the same?*”. Their findings highlight the potential differences in clot formation and stability between different fibrinogen concentrates, emphasizing the need for careful consideration when choosing treatment options.

Finally, Zhu et al. investigated “*Combined coagulation and inflammation markers as predictors of venous thrombo-embolism and death in COVID-19*” (10). Their study suggests that combining coagulation and inflammatory markers could refine prognostication of severe outcomes in COVID-19 patients.

The results of these studies demonstrate the complexity and diversity of the research on coagulation disorders and highlight the importance of continued efforts in this field to improve diagnosis, treatment, and patient outcomes across various coagulation-related conditions.

As host editors, we extend our sincere gratitude to the journal and its editors for their invaluable support in bringing this Research Topic of research to fruition and for the authors to submit their excellent manuscript to “*Frontiers in Medicine*”.

These findings not only contribute to the scientific knowledge base but also have the potential to significantly impact clinical practice, offering new hope for improved patient outcomes in the field of thrombosis and hemostasis.

We hope you enjoyed reading these ten articles as much as we did while preparing this edition. As host editors, it has been our pleasure to bring together this Research Topic of insightful works, and we trust they will inspire and inform your own endeavors in the field.

